# Case Report: Adequate T and B Cell Responses in a SARS-CoV-2 Infected Patient After Immune Checkpoint Inhibition

**DOI:** 10.3389/fimmu.2021.627186

**Published:** 2021-02-04

**Authors:** K. de Joode, A. A. M. Oostvogels, C. H. GeurtsvanKessel, R. D. de Vries, R. H. J. Mathijssen, R. Debets, A. A. M. van der Veldt

**Affiliations:** ^1^ Department of Medical Oncology, Erasmus MC Cancer Institute, Rotterdam, Netherlands; ^2^ Department of Viroscience, Erasmus Medical Center, Rotterdam, Netherlands; ^3^ Department of Radiology & Nuclear Medicine, Erasmus Medical Center, Rotterdam, Netherlands

**Keywords:** immunotherapy, adaptive immunity, cellular, humoral, kidney neoplasms, cancer

## Abstract

After the COVID-19 outbreak, non-evidence based guidelines were published to advise clinicians on the adjustment of oncological treatment during this pandemic. As immune checkpoint inhibitors directly affect the immune system, concerns have arisen about the safety of immunotherapy during this pandemic. However, data on the immune response in oncology patients treated with immunotherapy are still lacking. Here, we present the adaptive immune response in a SARS-CoV-2 infected patient who was treated with immune checkpoint inhibitors for advanced renal cell cancer. To evaluate the immune response in this patient, the number of T cells and their major subsets were measured according to expression of markers for co-signalling, maturation, and chemotaxis at baseline, during therapy, and during the SARS-CoV-2 infection. In addition, plasma samples were analyzed for IgM and IgG antibodies and the ability of these antibodies to neutralise SARS-CoV-2. Despite several risk factors for an impaired immune response to SARS-CoV-2, both T- and B-cell responses were observed. Moreover, after treatment with immune checkpoint inhibitors, a sufficient cellular and humoral immune response was achieved in this SARS-CoV-2 infected patient. These findings warrant renewed discussion on withholding of immune checkpoint inhibitors during an ongoing COVID-19 pandemic.

## Introduction

The coronavirus disease 2019 (COVID-19) pandemic, caused by severe acute respiratory syndrome coronavirus 2 (SARS-CoV-2), is having significant impact on oncological care. Besides capacity issues, concerns have arisen about the safety of oncological treatment and an increased risk for a more severe outcome of COVID-19 in patients with cancer (1–7). Patients with an (active) malignancy may have an increased risk of severe COVID-19, and it is still not known whether treatment with anti-cancer drugs—including immune checkpoint inhibitors (ICIs)—is safe during this pandemic (1–5).

After the first COVID-19 outbreak, non-evidence based guidelines were published to advise clinicians on the adjustment of oncological treatment during this pandemic. As ICIs directly affect the immune cells, and symptoms of COVID-19 resemble adverse events of ICIs (8), these guidelines were rather conservative regarding the use of ICIs during this pandemic. However, data on the immune response in patients infected with SARS-CoV-2 and treated with ICIs are still lacking (9). Here, we present for the first time data of the T and B cell responses in a SARS-CoV-2 infected patient who was treated with nivolumab and ipilimumab for advanced renal cell cancer (RCC).

## Case Description

In 2019, a 62-year-old male, with diabetes mellitus type II and hypertension, was diagnosed with primary metastatic RCC with lung and bone metastases. The disease was complicated by paraneoplastic pulmonary embolism for which therapeutic doses of low molecular weight heparin were started. Based on an interval of <1 year between diagnosis and systemic therapy, the patient had an “intermediate risk” according to the International Metastatic Renal Cell Carcinoma Database Consortium (IMDC) criteria. Therefore, first-line treatment with 3-weekly ipilimumab (1 mg/kg) plus nivolumab (3 mg/kg) was started while the primary tumor was *in situ (* 10). After four cycles of ipilimumab plus nivolumab, the first response evaluation showed progressive disease according to the Response Evaluation Criteria in Solid Tumours (RECIST) v1.1. However, as the patient experienced clinical benefit and some target lesions showed a reduction in tumor size with computed tomography (CT), maintenance treatment with nivolumab was started. After three cycles of 2-weekly maintenance treatment with nivolumab, creatinine levels increased from 126 µmol/L (i.e., baseline prior to ICI) to 265 µmol/L (>2x ULN) and the estimated glomerular filtration rate (eGFR) decreased from 53 ml/min (i.e., baseline prior to ICI) to 21 ml/min, which was accompanied by erythrocyturia and proteinuria. After the exclusion of other possible causes such as dehydration and contrast nephropathy, an immune related nephritis was considered most likely, although a biopsy to confirm this diagnosis could not be performed. According to the Common Terminology Criteria for Adverse Events Version 5 (CTCAE v5), the patient experienced a treatment-related nephritis grade 2 and treatment with steroids (prednisolone 1mg/kg) was started, whereas maintenance treatment with nivolumab was discontinued. In March 2020, the patient developed symptomatic COVID-19 with coughing and dyspnoea, infection with SARS-CoV-2 was confirmed by real-time polymerase chain reaction (RT-PCR). At the time of hospital admission (2 days after the onset of symptoms), the patient had been treated with prednisolone (1mg/kg daily) for four weeks and the last dose of nivolumab had been administered six weeks earlier. At that time, the creatinine level was decreased to 163 µmol/L with an eGFR of 38 ml/min. To prevent a potentially severe course of COVID-19, prednisolone was rapidly reduced to 60 mg daily within 3 days. As this dose reduction of prednisolone was accompanied by a grade 4 renal failure (creatinine level 500 µmol/L (>6.0 x ULN), eGFR 10 ml/min) according to CTCAE v5, high dose steroids (prednisolone 2 mg/kg daily, intravenously) was restarted. At hospital admission, empiric antibiotic treatment with cefuroxime and azithromycin was administered and minimal oxygen therapy was given for a few days. After 12 days of admission, the patient could be discharged. However, the patient was re-admitted within 7 days due to clinical deterioration. CT and magnetic resonance imaging (MRI) revealed extra- and intracranial progressive disease of RCC. MRI showed a newly diagnosed brain metastasis with bleeding. The patient experienced severe neurological deterioration and eventually died within 1 month after the first hospital admission for COVID-19. An autopsy was not performed.

## Immune Response

To study the adaptive immune response in this patient, peripheral blood samples were collected in the context of the MULTOMAB study (Netherlands Trial Registry number NL6828). In this observational study, blood samples are prospectively collected from patients with cancer treated with monoclonal antibodies. The MULTOMAB study has been approved by the medical ethics committee at Erasmus Medical Centre and the patient had signed informed consent. Blood samples were collected at baseline (prior to the first administration of ipilimumab plus nivolumab), 3 weeks after the first administration of ipilimumab plus nivolumab (T1), and during hospital admission for COVID-19 (23 weeks after the first administration of ipilimumab plus nivolumab;T2).

**Figure 1:**
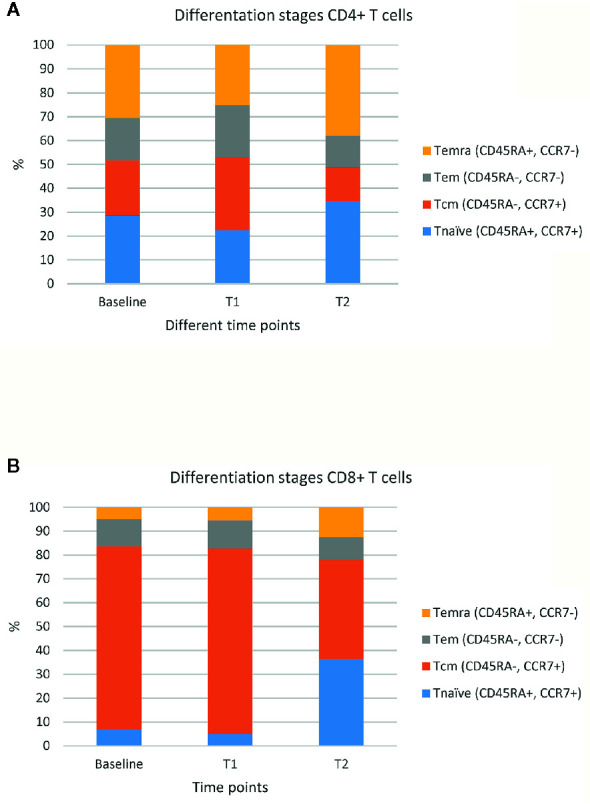
Percentages of different maturation stages of CD4+ and CD8+ T cells. Fractions of CD4+ T cells **(A)** and CD8+ T cells **(B)** in four differentiation stages at 3 time points: prior to ICIs (baseline), at 3 weeks after first administration of ipilimumab plus nivolumab (T1), and during SARS-CoV-2 infection (23 weeks after first administration of ipilimumab plus nivolumab; T2). The figure shows percentages of the following stages either within CD4+ and CD8+ T cells: naïve T cells (Tnaïve: CD45RA+, CCR7+), central memory T cells (Tcm: CD45RA-, CCR7+), effector memory T cells (Tem: CD45RA-, CCR7-), and effector memory T cells expressing CD45RA (Temra: CD45A+, CCR7-).

**Figure 2:**
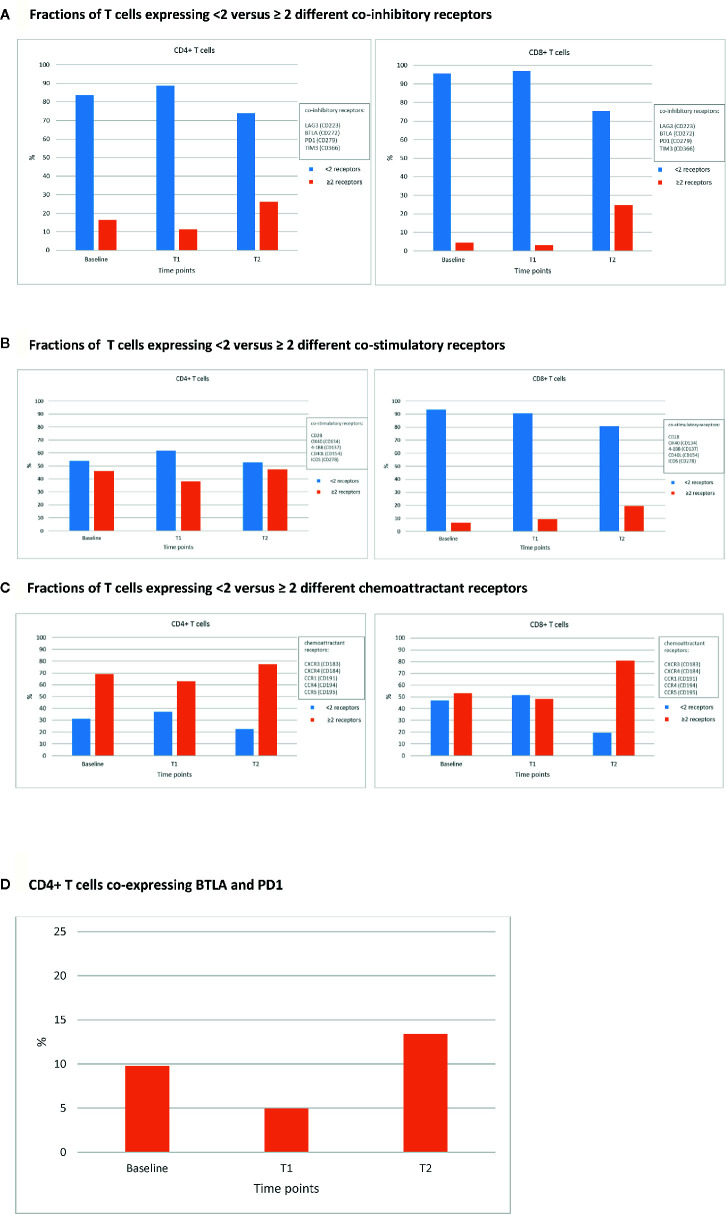
Percentages of CD4+ and CD8+ T cells expressing various classes of immune receptors. Fractions of CD4+ (left panels) and CD8+ (right panels) T cells expressing <2 versus ≥ 2 different co-inhibitory receptors. **(A)**, co-stimulatory receptors **(B)**, and chemoattractant receptors **(C)** at 3 different time points: prior to ICIs (baseline), at 3 weeks after first administration of ipilimumab plus nivolumab (T1), and during SARS-CoV-2 infection (23 weeks after first administration of ipilimumab plus nivolumab; T2). An example of the expression of co-inhibitory receptors is shown in **(D)**, where the fraction of CD4+ T cells co-expressing BTLA (CD272) and PD-1 (CD279) at baseline, T1, and T2 is shown.

After collection of these blood samples, peripheral blood mononuclear cells (PBMCs) and plasma were isolated for further analyses. Frequencies of T cells and their subsets prior to and during treatment with ICIs were determined by multiplex flow cytometry. The number of T cells and their major subsets were measured according to the expression of markers for co-signalling, maturation, and chemotaxis as previously described in detail (11). In addition, plasma samples were analyzed for IgM and total IgG antibodies directed against the receptor binding domain (RBD) of SARS-CoV-2 using an ELISA (Wantai) (12), and these antibodies were analyzed for their ability to neutralise SARS-CoV-2 by a plaque reduction test (PRNT50) (13). Upon diagnosis of COVID-19, the adaptive immune parameters changed drastically. First, the counts of total leukocytes showed a strong decrease from 6,9x10^3^/µl at T1 to 3.9 x10^3^/µl at T2, which was predominantly caused by a decrease in lymphocyte counts (**Supplementary Table 1**) **(** 14, 15). Second, for both CD4+ and CD8+ T cells the fractions of central (CD45RA-, CCR7+) and effector (CD45RA-, CCR7-) memory T cells decreased, whereas those of naïve T cells (CD45RA+, CCR7+) increased when comparing T2 versus T1 and baseline (**Figure 1**). As the total numbers of CD4+ and CD8+ T cells did not differ over time, the observed increase in naïve T cells may have been the result of apoptosis-mediated loss of more differentiated T cells. **Third**, the fractions of CD4+ and CD8+ T cells expressing multiple (≥2) types of co-inhibitory, co-stimulatory, and/or chemoattractant receptors increased when comparing T2 versus T1 (**Figures 2A–C**). The last two observations indicate that SARS-CoV-2 may have induced expression of multiple T cell receptors, which is often considered a measure of T cell differentiation. It is noteworthy that CD8+ T cells mainly expressed co-inhibitory and co-stimulatory receptors, whereas CD4+ T cells mainly expressed co-inhibitory receptors. In particular, fractions of CD4+ T cells expressing both programmed cell death protein 1 (PD-1) and B- and T-lymphocyte attenuator (BTLA) were high during infection with SARS-CoV-2 (**Figure 2D**). **Fourth**, the patient started to develop SARS-CoV-2 specific IgM and IgG antibodies at 10 days post onset of symptoms and neutralising antibodies were detectable at 15 days post onset of symptoms (**Supplementary Table 2**). Altogether, the above-mentioned T and B cell responses in this patient were assessed sufficient to clear the virus from the respiratory tract, as demonstrated by a negative virus culture at 15 days post onset of symptoms. The prolonged shedding of viral RNA is a phenomenon which is often observed, but does not necessarily indicate presence of infectious virus (16).

## Discussion

In this patient, who was infected with SARS-CoV-2 and treated with ICIs for metastatic RCC, an adequate cellular and humoral immune response was measured, despite the presence of risk factors for an impaired immune response and a severe course of SARS-CoV-2. For instance, an increased risk for a severe course of COVID-19 has been reported in patients who were treated with high-dose steroids prior to hospital admission (7). In addition, patients with cancer have an increased risk for a severe outcome of COVID-19, and this further increases in patients with progressive disease (6). Furthermore, this patient presented with lymphopenia, which is considered a predictive marker for severe COVID-19 (14, 15). In particular, the patient had an increased neutrophil-to-lymphocyte ratio (NLR)(6.5) and an increased lymphocyte-to-monocyte ratio (LMR) (5.3) which are also considered markers of poor survival in patients with COVID-19 (15). Finally, oncological guidelines stress the enhanced risk of treatment with ICIs during this pandemic (9, 17).

Besides the fact that this patient had several risk factors for an impaired immune response and severe course of COVID-19, adequate responses for both T and B cells were observed. T cell activation and consequently differentiation (**Figure 1**) may have led to loss of T cells, and an indirect increase in the fraction of naïve CD4+ and CD8+ T cells. The increased frequencies of T cells expressing immune receptors is another sign of T cell activation and differentiation (**Figure 2**). In particular, the enhanced frequency of CD4+ T cells expressing the co-inhibitory receptors BTLA and PD-1 (**Figure 2D**), is considered the result of an adaptive feedback loop to counter regulate the initial activation of T cells (18, 19). In addition to T cells, also B cell activation was observed by the production of neutralizing antibodies. As the viral culture was already negative despite low titers of antibodies measured with ELISA and PRNT50, the T cell response has most likely contributed to the clearance of the virus. Overall, both the presence of cellular as well as humoral immune parameters was comparable to those observed in SARS-CoV-2 infected patients without cancer (18–20).

As shown, the patients’ immune system was sufficiently active against SARS-CoV-2, but failed to act upon the renal cell cancer effectively. The latter deficit may be due to the existence of immune suppressive actions in the renal cell cancer micro-environment, preventing effective infiltration and/or activation of anti-tumor T cells (21). Already before COVID-19, the patient had progressive disease according to RECIST v.1.1, indicating that the renal cell cancer did not respond to immunotherapy. These results underscore different obstacles to achieve anti-tumor versus anti-virus immunity, and importantly demonstrate that, at least in this case, treatment with ICI does not alter the anti-virus T and B cell immunity.

Several studies have investigated the immune response to SARS-CoV-2 (18–20), and have yielded limited and conflicting data regarding COVID-19 in patients treated with ICIs (4, 9, 22). To the best of our knowledge, this is the first report on adaptive immunity in a SARS-CoV-2 infected patient treated with ICIs. Limitations of this report include the description of only one patient who had already discontinued treatment with ICIs. Although the patient discontinued treatment with ICIs at 6 weeks prior to the onset of COVID-19, it is conceivable that this treatment still affected the immune response. The ongoing effects of ICIs are well-known and are usually illustrated by their durable tumor response and late onset of adverse events, even months to years after discontinuation of treatment. In addition, specific measurements, such as NLR and LMR, were only performed during COVID-19 and could not be compared to previous values at baseline and during treatment with ICIs. The role of innate immune cells in this patient could not be elucidated in this particular case, and deserves further attention.

In conclusion, the adequate B and T cell responses in this SARS-CoV-2 infected patient who was treated with ICIs, justify renewed discussion on withholding of ICIs during the ongoing COVID-19 pandemic and may guide inclusion of patients treated with ICIs for COVID-19 vaccination (23).

## Data Availability Statement

The original contributions presented in the study are included in the article/**Supplementary Material**. Further inquiries can be directed to the corresponding author.

## Ethics Statement

The studies involving human participants were reviewed and approved by the MULTOMAB study (Netherlands Trial Registry number NL6828). Approved by medical ethics committee of Erasmus Medical Centre. The patients/participants provided their written informed consent to participate in this study. Written informed consent was obtained from the individual(s) for the publication of any potentially identifiable images or data included in this article.

## Author Contributions

KJ, AO, CG, RV and AV contributed to acquisition and analysis of the data. KJ and AV drafted the manuscript. RM and RD contributed to technical and material support. CG, RV, RM and RD contributed to critical revision of the manuscript for important intellectual content. All authors contributed to the article and approved the submitted version.

## Conflict of Interest

AV reports advisory board of BMS, MSD, Merck, Pfizer, Ipsen, Eisai, Pierre Fabre, Roche, Novartis, Sanofi, outside the submitted work. RM reports unrestricted grants for investigator-initiated research from Astellas, Bayer, Boehringer-Ingelheim, Cristal Therapeutics, Novartis, Pamgene, Pfizer, Roche, Sanofi, and Servier outside the submitted work. RD has received grants from Merck and Pan-Cancer T BV outside the submitted work.

The remaining authors declare that the research was conducted in the absence of any commercial or financial relationships that could be construed as a potential conflict of interest.
