# Supplementary material for: Case Report: Adequate T and B Cell Responses in a SARS-CoV-2 Infected Patient After Immune Checkpoint Inhibition
Source: Front Immunol. 2021 Feb 4;12:627186. doi: 10.3389/fimmu.2021.627186 (PMC7889602; doi:10.3389/fimmu.2021.627186)
Supplement: Supplementary file 1 [file Table_1.docx]

| **CD4+ T cells** |  | **Markers** | | **Baseline (%)** | | **T1**  **(%)** | **T2**  **(%)** |
| --- | --- | --- | --- | --- | --- | --- | --- |
|  |  |  |  |  |  |  |  |
|  | **maturation markers** | Tnaïve (CD45RA+, CCR7+) | | 29 | | 23 | 0 |
|  |  | Tcm (CD45RA-, CCR7+) | | 23 | | 31 | 14 |
|  |  | Tem (CD45RA-, CCR7-) | | 18 | | 22 | 13 |
|  |  | Temra (CD45RA+, CCR7-) | | 31 | | 25 | 38 |
|  | **co-inhibitory receptors** | CD39 | | 12 | | 11 | 16 |
|  |  | CD223 | | 1 | | 2 | 3 |
|  |  | CD272 | | 79 | | 61 | 88 |
|  |  | CD279 | | 12 | | 9 | 16 |
|  |  | CD366 | | 2 | | 2 | 3 |
|  | **co-stimulatory receptors** | CD28 | | 75 | | 69 | 81 |
|  |  | CD134 | | 0 | | 0 | 1 |
|  |  | CD137 | | 7 | | 8 | 4 |
|  |  | CD154 | | 30 | | 23 | 37 |
|  |  | CD278 | | 31 | | 26 | 28 |
|  | **chemoattractant receptors** | CD184 | | 100 | | 99 | 100 |
|  |  | CD191 | | 5 | | 4 | 6 |
|  |  | CD194 | | 36 | | 38 | 54 |
|  |  | CD195 | | 5 | | 5 | 2 |
|  |  | CXCR3 | 47 | | | 35 | 53 |
|  |  |  |  | | |  |  |
|  |  |  |  | | |  |  |
|  |  |  |  | | |  |  |
|  |  |  |  | | |  |  |
| **CD8+ T cells** |  | **Markers** | | | **Baseline (%)** | **T1**  **(%)** | **T2**  **(%)** |
|  |  |  |  |  |  |  |  |
|  | **maturation markers** | Tnaïve (CD45RA+, CCR7+) | | | 7 | 5 | 37 |
|  |  | Tcm (CD45RA-, CCR7+) | | | 77 | 78 | 42 |
|  |  | Tem (CD45RA-, CCR7-) | | | 11 | 12 | 10 |
|  |  | Temra (CD45RA+, CCR7-) | | | 5 | 5 | 13 |
|  | **co-inhibitory receptors** | CD39 | | | 1 | 1 | 12 |
|  |  | CD223 | | | 1 | 1 | 5 |
|  |  | CD272 | | | 62 | 39 | 74 |
|  |  | CD279 | | | 4 | 3 | 15 |
|  |  | CD366 | | | 2 | 2 | 6 |
|  | **co-stimulatory receptors** | CD28 | | | 20 | 22 | 31 |
|  |  | CD134 | | | 0 | 0 | 2 |
|  |  | CD137 | | | 3 | 3 | 4 |
|  |  | CD154 | | | 6 | 8 | 27 |
|  |  | CD278 | | | 6 | 6 | 12 |
|  | **chemoattractant receptors** | CD184 | | | 100 | 100 | 100 |
|  |  | CD191 | | | 28 | 23 | 43 |
|  |  | CD194 | | | 14 | 15 | 52 |
|  |  | CD195 | | | 18 | 20 | 40 |
|  |  | CXCR3 | | | 24 | 14 | 37 |

**Supplementary Table 1.** **Frequencies of different stages and functions of CD4+- and CD8+ T cells.**Frequency of CD4+ **(A)** and CD8+ **(B)** T cells that express particular maturation markers, or express a particular co-inhibitory, co-stimulatory or chemoattractant receptor at 3 time points: prior to ICI (baseline), at 3 weeks after first administration of ipilimumab plus nivolumab (T1), and during SARS-CoV-2 infection (i.e. 23 weeks after first administration of ipilimumab plus nivolumab; T2).
