# Supplementary material for: Case Report: Adequate T and B Cell Responses in a SARS-CoV-2 Infected Patient After Immune Checkpoint Inhibition
Source: Front Immunol. 2021 Feb 4;12:627186. doi: 10.3389/fimmu.2021.627186 (PMC7889602; doi:10.3389/fimmu.2021.627186)
Supplement: Supplementary file 2 [file Table_2.docx]

|  | **ELISA (Wantai)** | | | | **PRNT50** | | **RT-PCR throatswab** | | **Virus culture** |
| --- | --- | --- | --- | --- | --- | --- | --- | --- | --- |
| **Days post onset of COVID-19 symptoms** | **Ig (ratio)** | | **IgM (ratio)** | | **titer** |  | **CT ( E-gene)** | |  |
| 2 | 0 | neg | 0 | neg | <20 | neg | 16 | **pos** | not done |
| 4 | 0 | neg | 0 | neg | <20 | neg | 23 | **pos** | **pos** |
| 7 | 0,14 | neg | 0,05 | neg | <20 | neg | 19 | **pos** | **pos** |
| 10 | **6,27** | **pos** | **3,23** | **pos** | <20 | neg | 20 | **pos** | **pos** |
| 12 | **10,96** | **pos** | **4,85** | **pos** | <20 | neg | 21 | **pos** | **pos** |
| 15 | **8,45** | **pos** | **8,81** | **pos** | 20 | **pos** | 31 | **pos** | neg |

**Supplementary Table 2. Correlation of different diagnostics tests for SARS-CoV-2 infection at different time points during hospitalisation.**During hospitalisation diagnostic tests were performed at different time points post onset of COVID-19 symptoms. Plasma samples were analysed for IgM and total antibodies using Wantai ELISA, and the ability of these antibodies to neutralize SARS-CoV-2 was detected by a plaque reduction test (PRNT50). The cut-off for neutralizing antibodies is ≥20. A SARS CoV-2 specific RT-PCR was performed on throat swabs and a virus culture was performed on Vero cells.
